# Trends in Intention to Take the Second Booster COVID-19 Vaccination and Associated Factors in China: Serial Cross-Sectional Surveys

**DOI:** 10.3390/vaccines12050502

**Published:** 2024-05-07

**Authors:** Lingyu Kong, Xu Wang, Ziying Yang, Yihan Tang, Zhiwei Wang, Yu Ma, Jinghua Li, Zhoubin Zhang, Jing Gu

**Affiliations:** 1 Department of Medical Statistics, School of Public Health, Sun Yat-sen University, Guangzhou 510080, China; kongly8@mail2.sysu.edu.cn (L.K.); wangx766@mail2.sysu.edu.cn (X.W.); yangzy65@mail2.sysu.edu.cn (Z.Y.); tangyh39@mail2.sysu.edu.cn (Y.T.); lijinghua3@mail.sysu.edu.cn (J.L.); 2 Guangzhou Center for Disease Control and Prevention, Guangzhou 510440, Chinagzcdc_may@gz.gov.cn (Y.M.); 3 Sun Yat-sen Global Health Institute, School of Public Health and Institute of State Governance, Sun Yat-sen University, Guangzhou 510080, China; 4 Key Laboratory of Health Informatics of Guangdong Province, Sun Yat-sen University, Guangzhou 510080, China

**Keywords:** COVID-19, the second booster COVID-19 vaccination, vaccination intentions, psychological determinants, health behaviors

## Abstract

Background: The escalating complexity of the COVID-19 epidemic underscores the need for heightened attention to booster vaccinations. This study aims to examine the changing trend in the public’s intention to receive the second COVID-19 booster vaccination over time and the associated factors following the COVID-19 policy optimization in China. Method: Eight cross-sectional surveys utilizing SMS questionnaire links were conducted in Guangzhou, China, from December 2022 to April 2023. The Mann–Kendall test was employed to analyze the trend in intentions to receive the second booster vaccination across the survey time. Adjusted and multivariate logistic analyses were used to analyze the factors associated with vaccination intention. Parallel analyses were performed for two subgroups with different COVID-19 infection statuses. Results: A total of 9860 respondents were surveyed in the eight rounds, of which 8048 completed the first booster vaccination and were included in the analysis. The overall COVID-19 infection rate was 60.0% (4832/8048), while the overall vaccination intention was 72.2% (5810/8048) among respondents. The vaccination intention exhibited a significant declining trend over time, decreasing from 81.5% in December 2022 to 52.2% in April 2023. An adjusted logistic regression analysis revealed that anxiety and depression were negatively associated with an intention to receive the second booster vaccination, while COVID-19-related preventive behaviors and a high engagement in COVID-19-related information were positively associated with an intention to receive the second booster vaccination. A subgroup analysis revealed that the association between psychological and behavioral characteristics and vaccination intention remained relatively stable among individuals with different histories of COVID-19 infections. Conclusion: There was a significant decline in the intention to receive the second booster vaccination following the optimization of the COVID policy in China. Our findings emphasize the urgency of the second booster vaccination and provide a foundation for the development of tailored interventions to enhance and sustain vaccination intention among the public.

## 1. Introduction

The coronavirus 2019 (COVID-19) pandemic, caused by severe acute respiratory syndrome coronavirus 2 (SARS-CoV-2), remains a significant global public health concern. According to the World Health Organization (WHO), there have been more than 760 million confirmed cases and approximately seven million deaths globally as of June, 2023 [1]. The COVID-19 pandemic has also affected the healthcare system, such as hospital-acquired infections among patients [2]. With an enhanced understanding of COVID-19, vaccination is acknowledged as the most effective long-term strategy against the epidemic [3]. As of June 2023, approximately 13 billion COVID-19 vaccine doses have been administered worldwide [1]. In China, by June 2022, 91.74% of the population aged 3 years and above had completed the full primary COVID-19 vaccination, with 62.6% of those having received a booster vaccination [3].

As the protection of the initial booster dose of the COVID-19 vaccination diminishes, the immunity of residents has weakened alongside the emergence of SARS-CoV-2 variants with increased transmissibility and immune escape ability; different countries have launched the recommendation of administrating the second booster dose of the COVID-19 vaccine to the public [4,5,6,7,8]. For instance, individuals aged 50 and above with normal immune function are eligible for the second booster vaccination in the United States. The second booster for COVID-19 vaccination has shown its effectiveness in enhancing immunity and reducing the risk of COVID-19 infections [9], as well as decreasing the possibility of severe outcomes, such as hospitalization and mortality [6,8,10,11]. For example, a study conducted in the United States among people aged ≥ 50 found the effectiveness of preventing COVID-19-related hospitalizations increased from 55% to 80% after receiving the second booster vaccination [12]. Another nationwide observational study conducted in Hungary showed a 93% lower risk of COVID-19-related deaths in people who received two booster vaccinations compared to those who received only one booster vaccination during the Delta and Omicron waves [8].

COVID-19 vaccination intention could be influenced by various factors, such as the severity of the epidemic, types of vaccines, and an individual’s infection status. A cohort study in China revealed a substantial decline in an intention for immediate vaccination, decreasing from 58.3% during the well-contained phase to 23.0% during the severe epidemic phase [13]. A study conducted among American adults revealed that the type of COVID-19 vaccine influences people’s willingness to get vaccinated. This is primarily attributed to the varying efficacy, duration of protection, and advertised adverse reactions associated with different vaccine types [14]. Approximately 13.1% of individuals in Jordan exhibited reluctance to receive the COVID-19 booster vaccination because of a history of prior infection [15]. Moreover, individuals’ psychological and behavioral characteristics were also reported to be key factors regarding vaccination intention [16,17,18]. A survey conducted in the U.S. revealed that individuals who experienced depression and anxiety showed a decreased likelihood of receiving a COVID-19 booster vaccination [18]. Moreover, a study of 2053 Chinese factory workers found that COVID-19-related preventive behaviors, such as hand hygiene, wearing masks, and avoiding social gatherings, were positively associated with the intention of receiving a COVID-19 vaccination [16]. Another cross-sectional survey conducted in China showed that those who spent more time on social media obtaining COVID-19-related information were more likely to accept the second booster vaccination [17]. These psychological and behavioral factors reflect an individual’s intrinsic motivations to receive the vaccination. Identifying the factors associated with the second COVID-19 booster vaccination is essential to pinpoint the hesitant groups and develop evidence-based prevention and strategies aimed at prompting vaccination acceptance among the general public.

Since December 2022, the Chinese government has gradually optimized COVID-19 policy [19]. However, both the high infectivity of the virus and the relaxation of epidemic-related containment measures have resulted in the widespread transmission of COVID-19 across the country [20]. This further emphasizes the priority of the second booster immunization within the general population [21]. Given the unstable global pandemic situation and the continuous emergence of SARS-CoV-2 variants, an intention to receive COVID-19 vaccination may fluctuate across various stages of vaccination campaigns and the course of the epidemic [22,23]. Comprehending the trends and factors associated with intentions for the second booster COVID-19 vaccination is vital for insights into the evolving attitudes toward booster vaccination programs and the formulation of future vaccination strategies. However, there are limited studies available on the trend of acceptance rate and comprehensive associated factors to receive the second booster COVID-19 vaccination in China, especially in the early stage of change in epidemic control strategies.

In the current study, we investigated the temporal change in intention pertaining to the second booster COVID-19 vaccination since the optimization of the COVID policy. We then examined the association between health status, psychological and behavioral characteristics, survey time, and vaccination intention after adjusting for sociodemographic characteristics. Moreover, we explored the potential factors, such as psychological and behavioral characteristics associated with the vaccination intention. Given the fear related to the COVID-19 disease or the phenomenon known as “pandemic fatigue”, the history of COVID-19 infections may emerge as a crucial factor associated with vaccination intention [24]. Therefore, we further examined the differences in the factors associated with vaccination intention among individuals with different COVID-19 infection statuses.

## 2. Methods

### 2.1. Study Design and Population

This study used a repeated cross-sectional survey design, focusing on individuals aged 18 years and older who had been residing in Guangzhou for a minimum of six months. The online questionnaire survey was initiated on 22 November 2022 through the Guangzhou Health Hotline 12320, a nationwide official health service platform primarily involved in health education, promotion, and surveys [25]. The survey frequency was determined in accordance with the severity of the COVID-19 epidemic as follows: once per week in November and December 2022, once every two weeks in January 2023, and subsequently once per month. Questions related to booster vaccinations were included in the survey starting from 13 December 2022. Therefore, this study analyzed data from that date until April 2023. A total of 8 rounds of surveys were included in this study (13 December 2022, 20 December 2022, 27 December 2022, 6 January 2023, 15 January 2023, 8 February 2023, 9 March 2023, and 20 April 2023). The study obtained approval from the Ethics Committee of the School of Public Health, Sun Yat-sen University (No. 2020-005).

### 2.2. Data Collection

The epidemic risk personnel screening 12320 system was used to randomly select mobile phone numbers based on the population composition of each district in Guangzhou. In each survey round, 20,000 to 50,000 phone numbers were selected. Invitations via a Short Message Service (SMS) were sent to the selected phone numbers, providing information about the survey. Within the following three days, the survey link was sent to the respondents to collect information through an electronic questionnaire [26]. The questionnaire commenced by explaining the study’s objectives, contents, organizing institutions, and estimated time required to complete the questionnaire. Participants were informed that participation in the survey was voluntary and all personal information would be kept strictly confidential. Clicking to initiate the survey indicated informed consent to participate.

The click-through rate for each round of SMS invitations ranged from 4.6% to 12.5%, and the completion rate of questionnaires among those who opened the link ranged from 26.5% to 36.6%. The overall response rate (number of completed questionnaires/number of invitations sent) ranged from 3.5% to 5.5% in different rounds. Of the 9860 participants who completed the questionnaire, 8048 (81.6%) finished the first booster vaccination. As this study focused on the uptake of the second booster COVID-19 vaccination, the 8048 participants were included in the analysis.

## 3. Measurements

### 3.1. Primary Outcome

An intention to receive the second booster COVID-19 vaccination in the next six months was assessed among respondents who had completed the first booster vaccination. The intention was measured using binary options, namely “Yes” and “No” (Table A1). Respondents who responded “Yes” were defined as having a second booster vaccination intention.

### 3.2. Background Characteristics

Participants’ sociodemographic characteristics were collected, including sex (female and male), age (age group: 18-, 26-, 36-, 45-, 60-), place of residence (suburb and urban), marital status (unmarried, married, and divorced/widowed/other), and monthly income (Chinese yuan [CNY], <2000, 2001–5000, 5001–10,000, >10,000). Health characteristics included two items: whether one was diagnosed with chronic diseases before (yes and no) and whether one had been infected with COVID-19 since October 2022 (yes and no/not sure) (Table A1). We established December 2022 as the temporal boundary for our study as it aligned with China’s optimization of the COVID-19 prevention and control policies. Prior to this point, the infection rate of COVID-19 within the general population was notably low.

### 3.3. Psychological Characteristics

#### 3.3.1. The Generalized Anxiety Disorder Scale-7 (GAD-7)

The anxiety levels of the participants were assessed using the GAD-7 scale [27]. Respondents rated their anxiety symptoms over the past week (e.g., feeling nervous, anxious, or on edge) on a scale ranging from 0 = “Not at all” to 3 = “Nearly every day”. The total score ranges from 0 to 21, with a score of ≥10 indicating the presence of anxiety. The Cronbach’s alpha coefficient for this study was 0.963.

#### 3.3.2. Patient Health Questionnaire-9 (PHQ-9)

The depression levels of the participants were assessed using the PHQ-9 scale [28]. Respondents rated their depression symptoms over the past week (e.g., feeling easily annoyed or irritable) on a scale ranging from 0 = “Not at all” to 3 = “Nearly every day”. The total score ranges from 0 to 27, with a score of ≥10 indicating the presence of anxiety [29]. The Cronbach’s alpha coefficient for this study was 0.941.

#### 3.3.3. COVID-19-Related Worries

The assessment was self-constructed according to the literature, and it consisted of five items (Table A1) [30,31,32,33,34]. The respondents rated the extent of worries about the five given situations in the past week (e.g., oneself or family getting infected with COVID-19 and the COVID-19 pandemic affecting one’s or their family’s daily life). The five items were rated on a five-point scale from one = “Not worried at all” to five = “Very worried”. The total score ranged from 5 to 25, with the higher scores indicating more concerns to the COVID-19 epidemic. The Cronbach’s alpha coefficient for the five items was 0.904 in the present study.

## 4. Behavioral Characteristics

### 4.1. COVID-19-Related Preventive Behaviors

We employed three items to assess the level of COVID-19-related preventive behaviors among the respondents (i.e., wearing masks in public, washing hands immediately upon returning home, and maintaining a one-meter distance in queues) (Table A1). The three questions were generated according to the government’s behavioral instructions and literature [35,36,37]. The answer was a four-point scale, ranging from one = “not well-implemented [0–40% of time]” to four = “strictly implemented [91–100% of time]”. The total score of the measurement ranged from 3 to 12, with the higher score indicating a higher level of adherent protective behaviors. The Cronbach’s alpha coefficient for these three items was 0.746 in the present study.

### 4.2. COVID-19-Related Information Engagement Behavior

Information engagement is an active effort to purposefully obtain information from selected information carriers [38,39]. The level of COVID-19-related information engagement behavior was evaluated by one question: “Do you usually pay attention to information related to the pandemic on a daily basis?”. The answer was a four-point scale, ranging from one = “Not at all” to four = “Everyday”. Those who responded “sometimes” or “every day” were classified as high engagement, while those who answered “not at all” or “rarely” were classified as low engagement (Table A1).

### 4.3. Statistical Analysis

A descriptive analysis was performed by reporting frequencies and percentages for categorical variables, while the mean and standard deviation (SD) were used for continuous variables. Among those who finished the first booster vaccination, differences in the characteristics between respondents with or without an intention to receive the second booster vaccination were assessed using a *t*-test or Chi-square test. We used the Mann–Kendall test to assess the trend in intention to receive the second booster vaccination across the survey time. The associations between all studied variables and the second booster vaccination intention were assessed by univariate logistic regression models. An adjusted association analysis was performed to assess the association between health status, psychological and behavioral characteristics, survey time, and vaccination intention, adjusting for potential sociodemographic confounders with a *p*-value < 0.05 in the univariate analysis. Moreover, we further incorporated variables with statistical significance (*p*-value < 0.05) in the univariate analysis into the multivariate logistic regression model using the stepwise selection method to examine the factors associated with vaccination intention. The odds ratio (*OR*) and its 95% confidence interval (*CI*) were estimated. Similar analyses were performed for the two subgroups with different histories of COVID-19 infections parallelly. All statistical analyses were conducted using R 4.1.3 (R Foundation for Statistical Computing).

## 5. Results

### 5.1. Profiles of the Study Respondents

Profiles of the respondents who completed the survey (*n* = 9860) and were included in the analyses (finished the first booster vaccination, *n* = 8048) were similar (Table A2). As shown in Table 1, the mean age of the 8048 respondents was 36.3 ± 11.6 years old, and 56.6% of them were male. Over half of respondents resided in outlying urban areas (54.4%) and were married (54.2%). Approximately one-third of respondents reported a monthly income of 5001–10,000 CNY (33.4%). The sociodemographic characteristics of the respondents were similar across the eight waves of surveys (Table 1).

About 13.7% of respondents reported having a chronic disease, ranging from 12.7% to 16.5% across the serial surveys. In terms of psychological characteristics, 25.3% reported having experienced anxiety during the last week, increasing from 26.6% to 31.1% during the first three waves and then reducing to 14.4%; a total of 37.3% reported depression, increasing from 35.7% to 46.4% and then reducing to 22.9%. The mean score of worries related to COVID-19 was 16.0 ± 5.9. The level of worries related to COVID-19 remained stable during the initial five waves and was subsequently reduced to 13.1 ± 5.4. Regarding behavioral characteristics, the mean level of COVID-19-related preventive behavior was 10.3 ± 2.0. The level of preventive behavior remained stable during the initial five waves and subsequently decreased to 9.4 ± 2.2. The majority (85.8%) of respondents expressed high engagement with COVID-19-related information, and the percentage reduced to 52.2% across different waves of the surveys (Table 1).

### 5.2. Dynamics of Intention to Receive the Second Booster Vaccination and COVID-19 Infection Status during the Study Period

**Figure 1:**
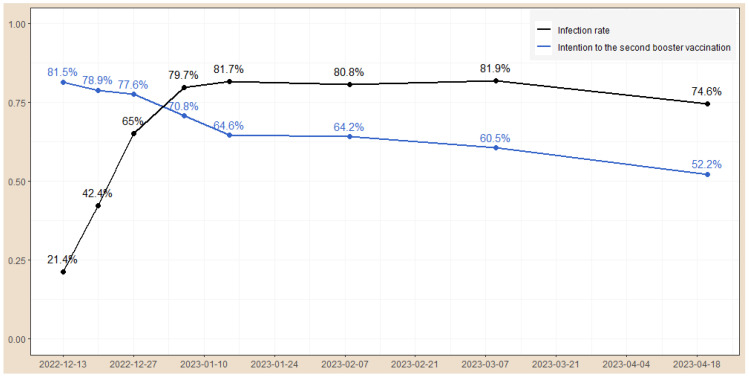
The temporal changes in COVID-19 infection rate and an intention to receive the second booster vaccination.

The overall rate of intention to receive the second booster vaccination among the study respondents was 72.2% (5810/8048) during the whole study period, and the declining trend over time was statistically significant (overall trend, *z*-value: −3.34, *p* <0.001, Figure 1). On 13 December 2022, approximately 81.5% of the respondents reported an intention to receive the second booster vaccination. The rate rapidly decreased to 64.6% on 15 January 2023 and to 52.2% on 20 April 2023. The overall infection rate of COVID-19 in our study sample was 60.0% (4832/8048). The infection rate increased from 21.4% on mid-December 2022, to 65% on 27 December 2022, peaked at 81.7% on January 2023, and remained relatively stable at around 80% after that (overall trend: *z*-value: 2.10, *p* = 0.035, Figure 1).

### 5.3. The Differences among Respondents with and without an Intention to Take the Second Booster Vaccination

As shown in Table 2, the average ages of the two groups were 35.3 ± 11.2 and 36.7 ± 11.7 years (*p* < 0.001), respectively. The group without an intention to take the second booster vaccination reported a higher proportion of being unmarried and having a monthly income < 2000 CNY compared to those with an intention (*p* < 0.05). The rate of COVID-19 infections was lower in the group with an intention to take the second booster compared to their counterparts (67.1% vs. 57.3%, *p* < 0.001). In terms of psychological and behavioral characteristics, the group with an intention to take the second booster had a lower percentage of anxiety (30.0% vs. 23.5%, *p* < 0.001) and depression (43.0% vs. 35.1%, *p* < 0.001). Furthermore, the group with an intention to take the second booster vaccination exhibited a higher level of worries related to COVID-19 (15.7 vs. 16.1, *p* = 0.232), higher scores of COVID-19-related preventive behaviors (9.7 vs. 10.5, *p* < 0.001), and higher engagement in COVID-19-related information (76.3% vs. 89.0%, *p* < 0.001) compared to their counterparts.

### 5.4. Factors Associated with an Intention to Receive the Second Booster Vaccination

After adjusting for potential background confounders (age, marital status, and monthly income) (Table 3), COVID-19 infections (*OR_a_* = 1.49, 95%CI: 1.35 to 1.66), high scores of worries about COVID-19 (*OR_a_* = 1.01, 95%CI: 1.00 to 1.02), high scores of preventive behaviors (*OR_a_* = 1.18, 95%CI: 1.15 to 1.21), and high engagement in COVID-19-related information (*OR_a_* = 2.45, 95%CI: 2.16 to 2.79) were positively associated with an intention to receive the second booster vaccination, while anxiety (*OR_a_* = 0.74, 95%CI: 0.66 to 0.83), depression (*OR_a_* = 0.74, 95%CI: 0.67 to 0.82), and survey time (*OR_a_* trend: *z*-value: −3.00, *p* = 0.003, Table 3) were negatively associated with vaccination intention. The multivariate logistic regression analysis, which incorporated variables exhibiting statistical significance (*p*-value < 0.05) in the univariate analysis, yielded similar results. The ultimate variables retained in the model encompassed anxiety (*OR_m_* = 0.78, 95%CI: 0.67 to 0.90), depression (*OR_m_* = 0.76, 95%CI: 0.67 to 0.87), COVID-19-related preventive behaviors (*OR_m_* = 1.09, 95%CI: 1.06 to 1.12), high engagement in COVID-19-related information (*OR_m_* = 1.84, 95%CI: 1.59 to 2.12), and survey time (*OR_m_* trend: *z*-value: −2.28, *p* = 0.022, Table 3).

### 5.5. Factors Associated with an Intention among Respondents with Different Infection Status

**Figure 2:**
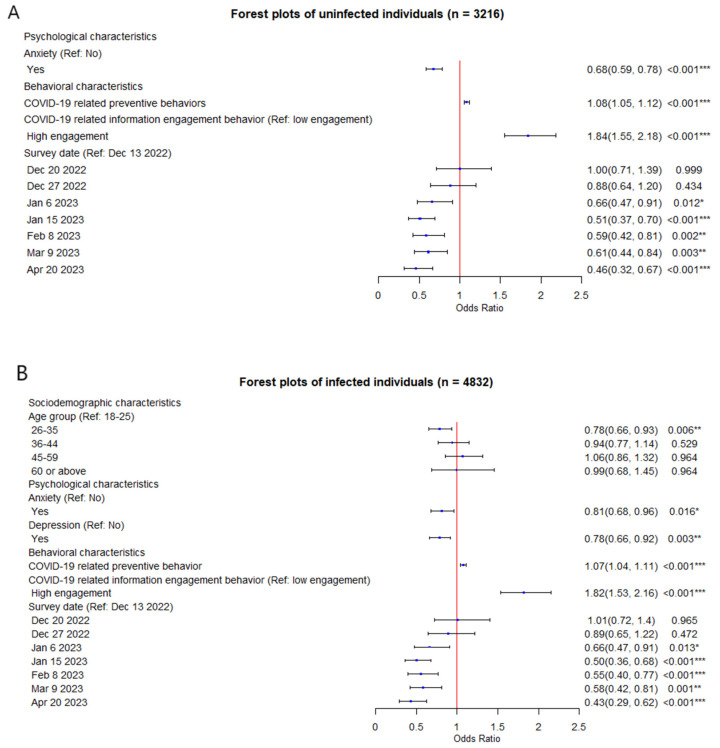
(**A**) Forest plot of potential factors associated with an intention to receive the second dose of COVID-19 vaccination among respondents without COVID-19 infection; (**B**) Forest plot of potential factors associated with an intention to receive the second dose of COVID-19 vaccination among respondents with COVID-19 infection. *: *p* < 0.05; **: *p* < 0.01; ***: *p* < 0.001.

Among the respondents who were uninfected with COVID-19, in the multivariate logistic regression analysis, variables that remained in the model included anxiety (*OR_m_* = 0.68, 95%CI: 0.59 to 0.78), COVID-19-related preventive behaviors (*OR_m_* = 1.08, 95%CI: 1.05 to 1.12), high engagement in COVID-19-related information (*OR_m_* = 1.84, 95%CI: 1.55 to 2.18), and survey time (*OR_m_* trend: *z*-value: −2.10, *p* = 0.035) (Table A3, Figure 2A). Among the respondents who had a history of COVID-19 infections, variables that remained in the final multivariate model included age group (26- vs. 18-, *OR_m_* = 0.78, 95%CI: 0.66 to 0.93), anxiety (*OR_m_* = 0.81, 95%CI: 0.68 to 0.96), depression (*OR_m_* = 0.78, 95%CI: 0.66 to 0.92), COVID-19-related preventive behaviors (*OR_m_* = 1.07, 95%CI: 1.04 to 1.11), high engagement in COVID-19-related information (*OR_m_* = 1.82, 95%CI: 1.53 to 2.16), and survey time (*OR_m_* trend: *z*-value: −2.10, *p* = 0.035) (Table A3, Figure 2B). Compared with the respondents who were uninfected with COVID-19, age and depression were negatively associated with vaccination intentions among respondents who had a history of COVID-19 infection.

## 6. Discussion

Based on the eight rounds of serial surveys conducted from December 2022 to April 2023, subsequent to China’s optimization of the COVID-19 policy in December 2022, our study found a noteworthy declining trend regarding an intention to receive the second booster vaccination and an increasing trend in the COVID-19 infection rate over this period. Psychological problems exhibited a negative association with vaccination intention, whereas the association between COVID-19-related preventive behaviors and vaccination intention was positively significant. Subgroup analyses were conducted on respondents, and differentiating between those who had been infected and those who had not been infected with COVID-19 yielded similar results.

Behavioral intention is a strong indicator of actual behavior [40]. In our study, a notably high initial intention (81.5%) was observed for individuals to receive the second booster vaccination upon the optimization of the COVID-19 policy in China. However, within the subsequent five months, this intention rate underwent a swift decline of approximately 30%, reaching 52.2%. The high intention to get the second booster vaccination may be related to the lower COVID-19 infection rate and higher infection risk in the population as of December 2022. As the COVID-19 infection rate in the general population continues to rise, residents naturally develop immunity to COVID-19, leading to a decrease in their perception of the risk of COVID-19 infections and, subsequently, a decline in their willingness to receive the second booster vaccination [41,42]. Meanwhile, since the Chinese government gradually optimized the zero-COVID policy in December 2022, the public’s attention to the epidemic has been gradually diminishing, potentially leading to pandemic fatigue as the situation develops [43,44]. Moreover, the diversity of second booster COVID-19 vaccine types, coupled with a lack of clear guidance, may also lead to a swift decline [14]. Although it is recommended to receive the next dose of the COVID-19 vaccination six months after the last vaccination [45], our study reveals a persistent decline in the public’s intention to continue receiving vaccinations. Following the optimization of prevention and control measures, and despite the existence of a specified duration of immune protection following COVID-19 contraction [41,42], the sustained decrease in booster vaccination intention emphasizes the necessity for proactive promotion of vaccinations within the general population.

In this study, depression and anxiety are negatively associated with an intention to receive the second booster vaccination. Similar findings were reported in previous studies [18,46,47]. For example, an internet study of adults in Latin America and the Caribbean found that respondents with depression were more reluctant to receive a booster dose of the COVID-19 vaccine [48]. A cross-sectional survey of adults in China found that the more mentally healthy people were, the more likely they were to receive the booster dose of the COVID-19 vaccine [49]. Individuals with psychological problems such as depression may encounter decision-making difficulties and tend to opt for retaining their current status, such as avoiding taking a vaccination [50]. The results indicated the influence of negative psychological well-being on vaccination intentions, underscoring the necessity for tailored screening and interventions to address the growing psychological support requirements of the general public during the epidemic. This could involve initiatives like enhanced online mental health education through the dissemination of educational articles and videos, as well as arranging sessions with mental health professionals for personalized psychological counseling and guidance [51].

Behavioral characteristics, including higher levels of COVID-19-related preventive behaviors and higher engagement with COVID-19-related information, were positively associated with vaccination intention [16,17]. Individuals who exhibit a higher degree of preventive behaviors, such as maintaining social distance, may possess stronger motivation and actions to safeguard themselves. Given the COVID-19 vaccination was perceived as the most effective measure to prevent infections, individuals who engaged more in COVID-19 preventive behaviors were more likely to express a higher intention to receive the second booster vaccination [52]. In terms of COVID-19-related information engagement behavior, active exposure to more COVID-19-related information on social media could enhance people’s knowledge and cognition of the COVID-19 epidemic and the second booster vaccination, subsequently reducing vaccine hesitancy and contribute to an increased rate of vaccination intention [17,53]. In accordance with the Delphi consensus to end the COVID-19 public health threat [54], our study suggests that authorities should implement measures, such as intensified vaccination propaganda and programs and enhanced dissemination of COVID-19-related knowledge, to strengthen public awareness and education regarding the necessity and effectiveness of the epidemic protective behaviors and information.

Notably, subgroup analysis revealed that the association between psychological and behavioral characteristics, survey time, and vaccination intentions remained relatively stable among individuals with different histories of COVID-19 infections. This finding further suggests that infection history may not be an important factor associated with vaccination intentions following the COVID-19 policy optimization in China. This departure from the conclusions drawn in prior studies signifies a notable shift in the dynamics surrounding second booster vaccination intentions [24,55]. According to the Information Motivation and Behavioral Skills (IMB) framework, individuals with improved vaccination knowledge could exhibit a higher level of intention to receive the second vaccination [56]. The absence of comprehensive promotional campaigns for the second booster vaccination, coupled with limited public health awareness, could potentially diminish individuals’ motivation and intentions to receive the second booster vaccination, irrespective of their COVID-19 infection history. Moreover, individuals with the mindset of acquiring sources and a heightened perception of the severity of epidemics are more likely to exhibit vaccination intentions [57]. The onset of pandemic fatigue and the reduced severity of COVID-19 variants may further exhaust the public’s interest in maintaining biosafety measures, including an intention to receive the second booster vaccination [44,58,59].

Our study has several limitations. First, the data, such as history of COVID-19 infections and behavioral characteristics, were collected through self-reported measures, which may be susceptible to recall bias or social desirability bias. Second, although numerous efforts were undertaken to conduct a wide-ranging survey, such as internet survey methods with SMS links and message reminders, the relatively low response rate and potential volunteer bias may limit the representativeness of our findings. Future efforts should be taken to increase the response rate, such as multiple reminders and incentives for participants. Third, our study had a serial cross-sectional design and thus could not determine the causal relationship between the variables. A longitudinal study was warranted to determine the dynamics of the relationships between the background variables and vaccination intentions. Fourth, the study population only included residents of Guangzhou, potentially resulting in an underrepresentation of the population from other areas of China. Fifth, due to the constraints of the survey duration, the study only investigated some key characteristics of the participants and did not include factors such as their level of education and occupation.

## 7. Conclusions

Our study examined the trend of intentions to receive the second booster of the COVID-19 vaccination and associated factors among the general population in China. We found that there was a significant decline in the intention to receive the second booster vaccination over time, from 81.5% to 52.2%. Psychological health status, COVID-19-related protective behaviors, COVID-19-related information engagement, and survey time were independently associated with an intention to receive the second booster vaccination. Our findings underscore the pressing need to enhance the public’s intention to receive the second booster vaccination and provide a foundation for targeted interventions.

## Figures and Tables

**Table 1:** The profiles of respondents across eight waves (*n*, %).

Characteristics	Overall	13 December 2022	20 December 2022	27 December 2022	6 January 2023	15 January 2023	8 February 2023	9 March 2023	20 April 2023
**Number of respondents**	8048	1382	1570	1456	801	913	718	806	402
**Sociodemographic characteristics**									
Sex (Female)	3495 (43.4)	611 (44.2)	668 (42.5)	666 (45.7)	330 (41.2)	420 (46.0)	290 (40.4)	336 (41.7)	174 (43.3)
Age (mean ± SD)	36.3 ± 11.6	35.7 ± 11.7	36.4 ± 11.0	36.5 ± 10.8	37.0 ± 11.7	36.6 ± 11.8	36.9 ± 12.6	34.9 ± 11.5	37.3 ± 13.0
Age group									
18-	1550 (19.3)	293 (21.2)	284 (18.1)	248 (17.0)	133 (16.6)	180 (19.7)	148 (20.6)	175 (21.7)	89 (22.1)
26-	2739 (34.0)	471 (34.1)	524 (33.4)	499 (34.3)	286 (35.7)	303 (33.2)	235 (32.7)	307 (38.1)	114 (28.4)
36-	1901 (23.6)	320 (23.2)	409 (26.1)	379 (26.0)	183 (22.8)	213 (23.3)	137 (19.1)	167 (20.7)	93 (23.1)
45-	1536 (19.1)	241 (17.4)	298 (19.0)	294 (20.2)	162 (20.2)	177 (19.4)	158 (22.0)	126 (15.6)	80 (19.9)
60-	322 (4.0)	57 (4.1)	55 (3.5)	36 (2.5)	37 (4.6)	40 (4.4)	40 (5.6)	31 (3.8)	26 (6.5)
Residential area (Urban)	3668 (45.6)	596 (43.1)	678 (43.2)	651 (44.7)	423 (52.8)	452 (49.5)	365 (50.8)	344 (42.7)	159 (39.6)
Marital status									
Unmarried	3179 (39.5)	585 (42.3)	580 (36.9)	550 (37.8)	304 (38.0)	348 (38.1)	286 (39.8)	359 (44.5)	167 (41.5)
Married	4365 (54.2)	713 (51.6)	890 (56.7)	811 (55.7)	438 (54.7)	505 (55.3)	390 (54.3)	407 (50.5)	211 (52.5)
Divorced/widowed/other	504 (6.3)	84 (6.1)	100 (6.4)	95 (6.5)	59 (7.4)	60 (6.6)	42 (5.8)	40 (5.0)	24 (6.0)
Monthly income (CNY)									
<2000 (<275 USD)	1514 (18.8)	261 (18.9)	320 (20.4)	282 (19.4)	148 (18.5)	162 (17.7)	124 (17.3)	140 (17.4)	77 (19.2)
2001- (275 USD-)	2172 (27.0)	414 (30.0)	468 (29.8)	366 (25.1)	205 (25.6)	238 (26.1)	187 (26.0)	206 (25.6)	88 (21.9)
5001- (685 USD-)	2686 (33.4)	433 (31.3)	513 (32.7)	499 (34.3)	281 (35.1)	308 (33.7)	248 (34.5)	267 (33.1)	137 (34.1)
10,001 (1370 USD-)	1676 (20.8)	274 (19.8)	269 (17.1)	309 (21.2)	167 (20.8)	205 (22.5)	159 (22.1)	193 (23.9)	100 (24.9)
**Health-related characteristics**									
Had chronic disease (Yes)	1105 (13.7)	175 (12.7)	204 (13.0)	197 (13.5)	105 (13.1)	151 (16.5)	104 (14.5)	115 (14.3)	54 (13.4)
Ever infected with COVID-19 (Yes)	4832 (60.0)	296 (21.4)	665 (42.4)	947 (65.0)	638 (79.7)	746 (81.7)	580 (80.8)	660 (81.9)	300 (74.6)
**Psychological characteristics**									
Anxiety (GAD-7, score ≥ 10)	2038 (25.3)	368 (26.6)	453 (28.9)	453 (31.1)	207 (25.8)	206 (22.6)	131 (18.2)	162 (20.1)	58 (14.4)
Depression (PHQ-9, score ≥ 10)	3005 (37.3)	494 (35.7)	645 (41.1)	675 (46.4)	337 (42.1)	334 (36.6)	196 (27.3)	232 (28.8)	92 (22.9)
Scores of worries about COVID-19	16.0 ± 5.9	17.0 ± 5.5	17.5 ± 5.6	17.6 ± 5.7	16.6 ± 5.4	16.0 ± 5.3	13.1 ± 5.4	11.8 ± 5.5	12.3 ± 5.2
**Behavioral characteristics**									
COVID-19-related preventive behavior	10.3 ± 2.0	10.8 ± 1.6	10.8 ± 1.6	10.8 ± 1.6	10.4 ± 1.9	10.2 ± 1.9	9.4 ± 2.2	8.7 ± 2.5	8.7 ± 2.4
COVID-19-related information engagement behavior (High engagement)	6880 (85.5)	1281 (92.7)	1442 (91.8)	1309 (89.9)	746 (93.1)	818 (89.6)	586 (81.6)	553 (68.6)	145 (36.1)
**Primary outcome**									
An intention to receive the second booster of COVID-19 vaccine (Yes)	5810 (72.2)	1126 (81.5)	1238 (78.9)	1130 (77.6)	567 (70.8)	590 (64.6)	461 (64.2)	488 (60.5)	210 (52.2)

Abbreviations: COVID-19: Coronavirus Disease 2019; GAD-7: General Anxiety Disorder-7; PHQ-9: Patient Health Questionnaire; SD: standard deviation.

**Table 2:** The difference of characteristics among respondents with and without an intention to take the second booster COVID-19 vaccination (*n* = 8048).

	Intention to Receive the Second Booster COVID-19 Vaccination (*n*, %)		*p*-Value
Characteristics	No (*n* = 2238)	Yes (*n* = 5810)	
**Sociodemographic characteristics**			
Sex (Female)			0.102
Female	1005 (44.9)	2490 (42.9)	
Age (mean ± SD)	35.3 ± 11.2	36.7 ± 11.7	<0.001 ***
Age group			
18-	449 (20.1)	1101 (19.0)	<0.001 ***
26-	855 (38.2)	1884 (32.4)	
36-	503 (22.5)	1398 (24.1)	
45-	347 (15.5)	1189 (20.5)	
60 or above	84 (3.8)	238 (4.1)	
Residence (Urban)	1023 (45.7)	2645 (45.5)	0.901
Marital status			
Unmarried	927 (41.4)	2252 (38.8)	0.032 *
Married	1189 (53.1)	3176 (54.7)	
Divorced/widowed or other	122 (5.5)	382 (6.6)	
Monthly income (CNY)			
<2000 (<275 USD)	446 (19.9)	1068 (18.4)	0.001 **
2001- (275 USD-)	574 (25.6)	1598 (27.5)	
5001- (685 USD-)	701 (31.3)	1985 (34.2)	
10,001 (1370 USD-)	517 (23.1)	1159 (19.9)	
**Health characteristics**			
Chronic disease (Yes)	291 (13.0)	814 (14.0)	0.254
Ever infected with COVID-19 (Yes)	1501 (67.1)	3331 (57.3)	<0.001 ***
**Psychological characteristics**			
Anxiety (GAD-7, score ≥ 10)	671 (30.0)	1367 (23.5)	<0.001 ***
Depression (PHQ-9, score ≥ 10)	963 (43.0)	2042 (35.1)	<0.001 ***
Scores of worries about COVID-19 (mean ± SD)	15.7 ± 6.1	16.1 ± 5.8	0.232
**Behavioral characteristics**			
COVID-19-related preventive behavior (mean ± SD)	9.7 ± 2.3	10.5 ± 1.9	<0.001 ***
COVID-19-related information engagement behavior (High engagement)	1707 (76.3)	5173 (89.0)	<0.001 ***

Abbreviations: COVID-19: Coronavirus Disease 2019; GAD-7: General Anxiety Disorder-7; PHQ-9: Patient Health Questionnaire; SD: standard deviation. *: *p* < 0.05; **: *p* < 0.01; ***: *p* < 0.001.

**Table 3:** Factors associated with an intention to receive the second booster COVID-19 vaccination.

Characteristics	Univariate Analysis		Adjusted Analysis		Multivariate Analysis	
	*OR_u_* (95%CI)	*p*-Value	*OR_a_* (95%CI)	*p*-Value	*OR_m_* (95%CI)	*p*-Value
**Sociodemographic characteristics**						
Sex (ref: male)						
Female	0.92 (0.83, 1.02)	0.096	N.A.		N.A.	
Age group (Ref: 18-)						
26-	0.90 (0.78, 1.03)	0.124	N.A.		N.S.	
36-	1.13 (0.98, 1.32)	0.101			N.S.	
45-	1.40 (1.19, 1.64)	<0.001 ***			N.S.	
60 or above	1.16 (0.88, 1.52)	0.298			N.S.	
Residential area (Ref: Suburb)			N.A.		N.A.	
Urban	0.99 (0.90, 1.10)	0.881				
Marital status (Ref: Unmarried)			N.A.			
Married	1.10 (0.99, 1.22)	0.067			N.S.	
Divorced/widowed or other	1.29 (1.04, 1.61)	0.022 *			N.S.	
Month income (Ref: <2000 CHY/275USD)			N.A.			
2001- (275 USD-)	1.16 (1.00, 1.35)	0.043 *			N.S.	
5001- (685 USD-)	1.18 (1.03, 1.36)	0.019 *			N.S.	
10,001 (1370 USD-)	0.94 (0.80, 1.09)	0.394			N.S.	
**Health characteristics**						
Chronic disease (Ref: No)						
Yes	1.09 (0.95, 1.26)	0.239	0.99 (0.85, 1.15)	0.882	N.A.	
Ever infected with COVID-19 (Ref: Yes)						
Uninfected/not sure	1.52 (1.37, 1.68)	<0.001 ***	1.49 (1.35, 1.66)	<0.001 ***	N.S.	
**Psychological characteristics**						
Anxiety (Ref: No)						
Yes	0.72 (0.65, 0.80)	<0.001 ***	0.74 (0.66, 0.83)	<0.001 ***	0.78 (0.67, 0.90)	<0.001 ***
Depression (Ref: No)						
Yes	0.72 (0.65, 0.79)	<0.001 ***	0.74 (0.67, 0.82)	<0.001 ***	0.76 (0.67, 0.87)	<0.001 ***
Scores of worries about COVID-19	1.01 (1.00, 1.02)	0.009 **	1.01 (1.00, 1.02)	0.001 **	N.S.	
**Behavioral characteristics**						
COVID-19-related preventive behavior	1.18 (1.16, 1.21)	<0.001 ***	1.18 (1.15, 1.21)	<0.001 ***	1.09 (1.06, 1.12)	<0.001 ***
COVID-19-related information engagement behavior (Ref: low engagement)						
High engagement	2.53 (2.22, 2.87)	<0.001 ***	2.45 (2.16, 2.79)	<0.001 ***	1.84 (1.59, 2.12)	<0.001 ***
**Survey date** (Ref: 13 December 2022)						
20 December 2022	0.85 (0.71, 1.02)	0.075	0.84 (0.70, 1.01)	0.061	0.86 (0.72, 1.03)	0.101
27 December 2022	0.79 (0.66, 0.95)	0.011 *	0.78 (0.65, 0.94)	0.009 **	0.83 (0.69, 1.00)	0.053
6 January 2023	0.55 (0.45, 0.68)	<0.001 ***	0.54 (0.44, 0.67)	<0.001 ***	0.57 (0.46, 0.70)	<0.001 ***
15 January 2023	0.42 (0.34, 0.50)	<0.001 ***	0.41 (0.34, 0.50)	<0.001 ***	0.43 (0.36, 0.53)	<0.001 ***
8 February 2023	0.41 (0.33, 0.50)	<0.001 ***	0.40 (0.32, 0.49)	<0.001 ***	0.46 (0.38, 0.57)	<0.001 ***
9 March 2023	0.35 (0.29, 0.42)	<0.001 ***	0.35 (0.29, 0.43)	<0.001 ***	0.46 (0.37, 0.57)	<0.001 ***
20 April 2023	0.25 (0.20, 0.32)	<0.001 ***	0.24 (0.19, 0.31)	<0.001 ***	0.38 (0.30, 0.50)	<0.001 ***

*: *p* < 0.05; **: *p* < 0.01; ***: *p* < 0.001. *OR_u_*: the OR values of univariate logistic regression analysis; *OR_a_:* the OR values after adjusting for sociodemographic variables with *p* < 0.05 in univariate analysis (i.e., age, marital status and monthly income). *OR_m_*: the OR values of multivariate logistic analysis (stepwise) including all variables. N.S.: non-significant. N.A.: not applicable.

## Data Availability

Data are contained within the article.

## Appendix A

**Table A1:** Questionnaire structure of primary outcome, psychological and behavioral information.

Items	
**Intention to receive the second booster vaccination**	
Will you receive a second booster dose of COVID-19 vaccine in the next six months?	0: No; 1: Yes
**Psychological information**	
**COVID-19-related worries**	
In the past week, to what extent have you been worried about COVID-19 infection for yourself and your family members?	1: Not worried at all; 2: Not very worried; 3: Somewhat worried; 4: Worried; 5: Very worried
In the past week, to what extent have you been worried about the impact of the pandemic on the daily lives of you and your family members?	1: Not worried at all; 2: Not very worried; 3: Somewhat worried; 4: Worried; 5: Very worried
There may be a large number of new COVID-19 cases in Guangzhou in the future	1: Not worried at all; 2: Not very worried; 3: Somewhat worried; 4: Worried; 5: Very worried
There may be a significant number of COVID-19 deaths in Guangzhou in the future	1: Not worried at all; 2: Not very worried; 3: Somewhat worried; 4: Worried; 5: Very worried
The healthcare system may face challenges in coping with the situation in the future system	1: Not worried at all; 2: Not very worried; 3: Somewhat worried; 4: Worried; 5: Very worried
**Behavioral information**	
**COVID-19-related preventive behaviors**	
Wearing masks in public	1: not well-implemented [0–40% of time]; 2: partially implemented [41–70% of time]; 3: mostly implemented [71–90% of time]; 4: strictly implemented [91–100% of time]
Washing hands immediately upon returning home	1: not well-implemented [0–40% of time]; 2: partially implemented [41–70% of time]; 3: mostly implemented [71–90% of time]; 4: strictly implemented [91–100% of time]
Maintaining a one-meter distance in line	1: not well-implemented [0–40% of time]; 2: partially implemented [41–70% of time]; 3: mostly implemented [71–90% of time]; 4: strictly implemented [91–100% of time]
**COVID-19-related information engagement behavior**	
Do you usually pay attention to information related to the pandemic on a daily basis?	1: Not at all; 2: Rarely; 3: Sometimes; 4: Everyday

**Table A2:** The profiles of the respondents who completed the survey and included in the analysis (*n*, %).

Characteristics	Respondents Completed the Survey	Respondents Included in the Analyses	*p*-Value
**Number of respondents**	9860	8048	
**Sociodemographic characteristics**			
Sex (Female)	4363 (44.2)	3495 (43.4)	0.270
Age (mean ± SD)	36.1 ± 11.6	36.3 ± 11.6	0.251
Age group			0.277
18-	1923 (19.5)	1550 (19.3)	
26-	3473 (35.2)	2739 (34.0)	
36-	2265 (23.0)	1901 (23.6)	
45-	1787 (18.1)	1536 (19.1)	
60-	406 (4.1)	322 (4.0)	
Residential area (Urban)	4541 (46.1)	3668 (45.6)	0.523
Marital status			0.579
Unmarried	3934 (39.9)	3179 (39.5)	
Married	5281 (53.6)	4365 (54.2)	
Divorced/widowed/other	645 (6.5)	504 (6.3)	
Monthly income (CNY)			0.173
<2000 (<275 USD)	1904 (19.3)	1514 (18.8)	
2001- (275 USD-)	2546 (25.8)	2172 (27.0)	
5001- (685 USD-)	3260 (33.1)	2686 (33.4)	
10,001 (1370 USD-)	2150 (21.8)	1676 (20.8)	
**Health characteristics**			
Chronic disease (Yes)	1427 (14.5)	1105 (13.7)	0.156
Ever infected with COVID-19 (Yes)	5886 (59.7)	4832 (60.0)	0.640
**Psychological characteristics**			
Anxiety (GAD-7, score ≥ 10)	2604 (26.4)	2038 (25.3)	0.099
Depression (PHQ-9, score ≥ 10)	3771 (38.2)	3005 (37.3)	0.213
Scores of worries about COVID-19	16.0 ± 5.9	16.0 ± 5.9	0.536
**Behavioral characteristics**			
COVID-19-related preventive behavior	12.6 ± 2.9	10.3 ± 2.0	<0.001 ***
COVID-19-related information engagement behavior (High engagement)	8353 (84.7)	6880 (85.5)	0.150
**Primary outcome**			
An intention of the second booster of COVID-19 vaccine (Yes)		5810 (72.2)	

Abbreviations: COVID-19, Coronavirus Disease 2019; GAD-7, General Anxiety Disorder-7; PHQ-9, Patient Health Questionnaire; SD: standard deviation. ***: *p* < 0.001.

**Table A3:** Multivariable logistic regression analysis of potential factors associated with the willingness to receive the second dose of COVID-19 vaccination among respondents with different infection status.

Characteristics	Uninfected				Infected			
	*OR_u_* (95% CI)	*p*-Value	*OR_m_* (95%CI)	*p*-Value	*OR_u_* (95% CI)	*p*-Value	*OR_m_* (95%CI)	*p*-Value
**Sociodemographic information**								
Sex (Ref: male)							N.A.	
Female	0.93 (0.83, 1.05)	0.260	N.A.		1.00 (0.84, 1.18)	0.980		
Age group (Ref: 18-)								
26-	0.86 (0.73, 1.02)	0.087	N.S.		0.99 (0.78, 1.24)	0.906	0.78 (0.66, 0.93)	0.006 **
36-	1.14 (0.95, 1.38)	0.170	N.S.		1.10 (0.86, 1.42)	0.444	0.94 (0.77, 1.14)	0.529
45-	1.34 (1.09, 1.64)	0.005 **	N.S.		1.46 (1.11, 1.91)	0.007 **	1.06 (0.86, 1.32)	0.964
60 or above	1.23 (0.86, 1.78)	0.264	N.S.		1.00 (0.67, 1.53)	0.992	0.99 (0.68, 1.45)	0.964
Residential area (Ref: Suburb)							N.A.	
Urban	1.14 (1.01, 1.29)	0.030	N.A.		0.88 (0.75, 1.04)	0.140		
Marital status (Ref: Unmarried)							N.A.	
Married	1.09 (0.96, 1.23)	0.201	N.S.		1.13 (0.95, 1.34)	0.170		
Divorced/widowed or other	1.43 (1.07, 1.92)	0.016 *	N.S.		1.06 (0.77, 1.49)	0.720		
Month income (Ref: <2000 CHY/275USD)							N.A.	
2001- (275 USD-)	1.19 (0.98, 1.43)	0.072	N.S.		1.14 (0.90, 1.43)	0.288		
5001- (685 USD-)	1.20 (1.00, 1.43)	0.047 *	N.S.		1.25 (0.99, 1.58)	0.056		
10,001 (1370 USD-)	1.00 (0.83, 1.21)	0.963	N.S.		0.91 (0.71, 1.18)	0.472		
Chronic disease (Ref: No)							N.A.	
Yes	1.26 (1.05, 1.53)	0.014 *	N.S.		0.84 (0.67, 1.05)	0.130	N.A.	
**Psychological information**								
Anxiety (Ref: No)								
Yes	0.76 (0.66, 0.87)	<0.001 ***	0.68 (0.59, 0.78)	<0.001 ***	0.68 (0.57, 0.83)	<0.001 ***	0.81 (0.68, 0.96)	0.016 *
Depression (Ref: No)								
Yes	0.76 (0.68, 0.87)	<0.001 ***	N.S.		0.71 (0.59, 0.84)	<0.001 ***	0.78 (0.66, 0.92)	0.003 **
Scores of worries about COVID-19	1.00 (0.99, 1.02)	0.360	N.A.		1.02 (1.00, 1.03)	0.019 *	N.S.	
**Behavioral information**								
COVID-19-related preventive behavior	1.15 (1.12, 1.19)	<0.001 ***	1.08 (1.05, 1.12)	<0.001 ***	1.21 (1.16, 1.26)	<0.001 ***	1.07 (1.04, 1.11)	<0.001 ***
COVID-19-related information engagement behavior (Ref: low engagement)								
High engagement	2.28 (1.96, 2.66)	<0.001 ***	1.84 (1.55, 2.18)	<0.001 ***	2.81 (2.22, 3.55)	<0.001 ***	1.82 (1.53, 2.16)	<0.001 ***
**Survey date** (Ref: 13 December 2022)								
20 December 2022	1.00 (0.72, 1.39)	0.989	1.00 (0.71, 1.39)	0.999	0.83 (0.66, 1.04)	0.107	1.01 (0.72, 1.4)	0.965
27 December 2022	0.88 (0.64, 1.19)	0.418	0.88 (0.64, 1.20)	0.434	0.95 (0.72, 1.25)	0.705	0.89 (0.65, 1.22)	0.472
6 January 2023	0.67 (0.48, 0.92)	0.015 *	0.66 (0.47, 0.91)	0.012 *	0.59 (0.41, 0.87)	0.007 **	0.66 (0.47, 0.91)	0.013 *
15 January 2023	0.51 (0.37, 0.69)	<0.001 ***	0.51 (0.37, 0.70)	<0.001 ***	0.44 (0.31, 0.64)	<0.001 ***	0.50 (0.36, 0.68)	<0.001 ***
8 February 2023	0.54 (0.39, 0.74)	<0.001 ***	0.59 (0.42, 0.81)	0.002 **	0.32 (0.22, 0.47)	<0.001 ***	0.55 (0.40, 0.77)	<0.001 ***
9 March 2023	0.48 (0.35, 0.66)	<0.001 ***	0.61 (0.44, 0.84)	0.003 **	0.22 (0.16, 0.32)	<0.001 ***	0.58 (0.42, 0.81)	0.001 **
20 April 2023	0.31 (0.21, 0.44)	<0.001 ***	0.46 (0.32, 0.67)	<0.001 ***	0.25 (0.16, 0.38)	<0.001 ***	0.43 (0.29, 0.62)	<0.001 ***

Abbreviations: COVID-19, Coronavirus Disease 2019; GAD-7, General Anxiety Disorder-7; N.A.: not applicable, N.S.: no statistical; PHQ-9, Patient Health Questionnaire; SD, standard deviation; *: *p* < 0.05; **: *p* < 0.01; ***: *p* < 0.001. *OR_u_*: the OR values of univariate logistic analysis; *OR_m_*: the OR values of multivariate logistic analysis. N.S.: non-significant.
